# Establishment of a New Quantitative Evaluation Model of the Targets’ Geometry Distribution for Terrestrial Laser Scanning

**DOI:** 10.3390/s20020555

**Published:** 2020-01-19

**Authors:** Ronghua Yang, Xiaolin Meng, Zejun Xiang, Yingmin Li, Yangsheng You, Huaien Zeng

**Affiliations:** 1 Key Laboratory of New Technology for Construction of Cities in Mountain Area (Chongqing University), Ministry of Education, Chongqing 400045, China; liyingmin@cqu.edu.cn (Y.L.); youyangsheng@126.com (Y.Y.); 2 School of Civil Engineering, Chongqing University, Chongqing 400045, China; 3 Chongqing Survey Institute, Chongqing 401121, China; xiangzj@cqkcy.com; 4 Nottingham Geospatial Institute, The University of Nottingham, Nottingham NG7 2TU, UK; xiaolin.meng@nottingham.ac.uk; 5 Hubei Key Laboratory of Intelligent Vision Based Monitoring for Hydroelectric Engineering, China Three Gorges University, Yichang 443002, China; zenghuaien_2003@163.com; 6 Key Laboratory of Geological Hazards on Three Gorges Reservoir Area (China Three Gorges University), Ministry of Education, Yichang 443002, China

**Keywords:** terrestrial laser scanning, target-based registration, targets’ geometry distribution, precision of the registration

## Abstract

The precision of target-based registration is related to the geometry distribution of targets, while the current method of setting the targets mainly depends on experience, and the impact is only evaluated qualitatively by the findings from empirical experiments and through simulations. In this paper, we propose a new quantitative evaluation model, which is comprised of the rotation dilution of precision (rDOP, assessing the impact of targets’ geometry distribution on the rotation parameters) and the translation dilution of precision (tDOP, assessing the impact of targets’ geometry distribution on the translation parameters). Here, the definitions and derivation of relevant formulas of the rDOP and tDOP are given, the experience conclusions are theoretically proven by the model of rDOP and tDOP, and an accurate method for determining the optimal placement location of targets and the scanner is proposed by calculating the minimum value of rDOP and tDOP. Furthermore, we can refer to the model (rDOP and tDOP) as a unified model of the geometric distribution evaluation model, which includes the DOP model in GPS.

## 1. Introduction

Terrestrial laser scanning (TLS) can provide a three-dimensional (3D) spatial point cloud dataset of the objects’ surfaces. The spatial resolution of the data is much higher than that of conventional surveying methods [1]. Due to occluded surfaces and limitations in the view of a scanner, we usually need to make several scans from different setups of the scanner in order to survey a quite large and complex object [2,3]. These point clouds (scans) must first be registered to a chosen coordinate system before a coherent parametric description of the object can be formed [4]. Target-based registration with two scans is one of the most common registration approaches and is often performed using a 3D rigid body transformation algorithm [5,6]. 

Although target-based registration technology is relatively mature [7], studies of the target-based registration precision are still required, such as those on measurement improvement [8,9], the uncertainty of the target center estimation [10], the error propagation for two scans and multiple scans [3,4,11], the directly geo-referenced TLS data precision [3,12], the relationship between the registration precision and the rotation and translation matrices [13], the relationship between the registration precision and the targets’ geometry distribution (TGD) [2], etc. For the relationship between the registration precision and TGD, some researchers have found that an increase in the number of targets can improve the registration precision, and think that the targets will be distributed evenly and will not lie on the same line or be close to such a configuration [2,14,15,16,17,18,19,20]. Fan et al. [12] and Liu et al. [17] used a simulation method to demonstrate that the registration error is inversely proportional to the number of targets and the sum of distances between targets and the barycenter of all targets. Bornaz et al. [21] proved that the registration precision of two scans depends on the overlap ratio adopted (namely the target distribution range of the overlap area), and found that the minimum overlap ratio of 30% is required for assuring a final precision comparable to the range precision of the used instruments. However, all of the above studies have only evaluated the impact of TGD on the registration precision qualitatively through empirical experiments and simulations, while there has been no research conducted on the theoretical evaluation model for it. As a result, we can never know the best location of the scanner and the best TGD. How can we quantitatively evaluate the impact of TGD and describe the relationship between TGD and registration precision? These issues are the focus of this research.

In this study, we first used the theorem of error propagation to constitute a new theoretical evaluation model of the TGD, that is, the rotation dilution of precision (rDOP) and the translation dilution of precision (tDOP); we then theoretically analyzed the model’s existence conditions, the relationship between the model and the number of targets, and the model’s bounds; and finally, we verified the evaluation model of the TGD by conducting experiments.

## 2. Methods

There are two kinds of situations in practical applications, which are “we need to determine the optimal setting position of scanner where TGD is known” and “we need to determine the optimal TGD where the position of scanner is known”. The unit of the rotation parameters is different from the unit of translation parameters, and the calculation results of translation and rotation will interact with each other when the transformation parameters are dependent on calculation models. For these reasons, we will first introduce the common registration model of two scans. We will then present the calculation of rotation parameters using the Rodrigues matrix [3,6]. Thirdly, we will propose a new calculation method of the translation parameter (similar to spatial distance resection in GPS [22]), which can ensure that the parameters of translation and rotation are computed independently. Fourthly, we will propose a new quantitative evaluation model of TGD, namely rDOP (which can be used to help determine the optimal TGD) and tDOP (which can be used to help determine the optimal setting position of scanner). Finally, we will derive an equal weight model of TGD and propose a set of model application schemes.

### 2.1. Registration Model of Two Scans

In the context of TLS, registration is the transformation of multiple point clouds (scans) into the coordinate system of a chosen scan [2]. The rigid body transformation operation of registration is expressed in Equation (1), in which the point clouds in ***Scan i* + 1** are transformed into ***Scan i*** using the three translation parameters $t_{x}$, $t_{y}$, and $t_{z}$ and the three rotation parameters $r_{a}$, $r_{b}$, and $r_{c}$ [3,23].

(1) $$p_{j}^{i}=\left[\begin{array}{c} x_{j}^{i}\\ y_{j}^{i}\\ z_{j}^{i} \end{array}\right]=R\left[\begin{array}{c} x_{j}^{i+1}\\ y_{j}^{i+1}\\ z_{j}^{i+1} \end{array}\right]+T=Rp_{j}^{i+1}+T,$$

where $p_{j}^{i}$ and $p_{j}^{i+1}$ represent the same target in ***Scan i*** and ***Scan i* + 1**, respectively, whose observation values of coordinates are $\left(x_{j}^{i},y_{j}^{i},z_{j}^{i}\right)$ and $\left(x_{j}^{i+1},y_{j}^{i+1},z_{j}^{i+1}\right)$; R is the standard 3×3 rotation matrix; T is the 3×1 translation vector; and

(2) $$R=\frac{1}{1+r_{a}^{2}+r_{b}^{2}+r_{c}^{2}}\left[\begin{array}{ccc} 1+r_{a}^{2}-r_{b}^{2}-r_{c}^{2} & 2(r_{c}+r_{a}r_{b}) & 2(r_{a}r_{c}-r_{b})\\ 2(r_{a}r_{b}-r_{c}) & 1-r_{a}^{2}+r_{b}^{2}-r_{c}^{2} & 2(r_{a}+r_{b}r_{c})\\ 2(r_{b}+r_{a}r_{c}) & 2(r_{b}r_{c}-r_{a}) & 1-r_{a}^{2}-r_{b}^{2}+r_{c}^{2} \end{array}\right],\;T=\left[\begin{array}{c} t_{x}\\ t_{y}\\ t_{z} \end{array}\right].$$

For uniquely determining the above transformation parameters between ***Scan i*** and ***Scan Scan i* + 1**, we usually need to use three or more targets with known 3D coordinates [2,21], and these targets are placed in the overlap locations between the two point-clouds. 

In this study, we assumed that the number of targets k is greater than 3 (k≥3) and the coordinate of any point in ***Scan i*** (on a chosen coordinate system) is known, and we employed scanning to obtain the new point cloud in ***Scan Scan i* + 1**, which was transformed into ***Scan i***.

### 2.2. Calculation of Rotation Parameters

If $p_{jc}^{i}=p_{j}^{i}-\frac{1}{k}\sum_{j_{m}=1}^{k}p_{j_{m}}^{i}$ and $p_{jc}^{i+1}=p_{j}^{i+1}-\frac{1}{k}\sum_{j_{m}=1}^{k}p_{j_{m}}^{i+1}$, with Equation (1), we can get

(3) $$p_{jc}^{i}=\left[\begin{array}{c} x_{jc}^{i}\\ y_{jc}^{i}\\ z_{jc}^{i} \end{array}\right]=R\left[\begin{array}{c} x_{jc}^{i+1}\\ y_{jc}^{i+1}\\ z_{jc}^{i+1} \end{array}\right]=Rp_{jc}^{i+1}.$$

From the Rodrigues matrix [21,23], with Equations (2) and (3), we can get

(4) $$(I_{3}+S)\left[\begin{array}{c} x_{jc}^{i}\\ y_{jc}^{i}\\ z_{jc}^{i} \end{array}\right]=(I_{3}-S)\left[\begin{array}{c} x_{jc}^{i+1}\\ y_{jc}^{i+1}\\ z_{jc}^{i+1} \end{array}\right],$$

where $S=\left[\begin{array}{ccc} 0 & -r_{c} & r_{b}\\ r_{c} & 0 & -r_{a}\\ -r_{b} & r_{a} & 0 \end{array}\right]$ and $R={\left(I_{3}+S\right)}^{-1}(I_{3}-S)$.

With Equation (4), we can get

(5) $$\alpha_{j}\left[\begin{array}{c} r_{a}\\ r_{b}\\ r_{c} \end{array}\right]=p_{jc}^{i}-p_{jc}^{i+1},\;j=1,2,\cdots ,k,$$

where $S=\left[\begin{array}{ccc} 0 & -r_{c} & r_{b}\\ r_{c} & 0 & -r_{a}\\ -r_{b} & r_{a} & 0 \end{array}\right]$ and $R={\left(I_{3}+S\right)}^{-1}(I_{3}-S)$.

(6) $$\alpha_{j}=\left[\begin{array}{ccc} 0 & -\tau_{z,j} & \tau_{y,j}\\ \tau_{z,j} & 0 & -\tau_{x,j}\\ -\tau_{y,j} & \tau_{x,j} & 0 \end{array}\right],\;\left[\begin{array}{c} \tau_{x,j}\\ \tau_{y,j}\\ \tau_{z,j} \end{array}\right]=\left[\begin{array}{c} x_{jc}^{i}+x_{jc}^{i+1}\\ y_{jc}^{i}+y_{jc}^{i+1}\\ z_{jc}^{i}+z_{jc}^{i+1} \end{array}\right]=p_{jc}^{i}+p_{jc}^{i+1}=(I_{3}+R)p_{jc}^{i}.$$

If the estimated values of $r_{a}$, $r_{b}$, and $r_{c}$ are ${\hat{r}}_{a}$, ${\hat{r}}_{b}$, and ${\hat{r}}_{c}$, respectively, with Equations (5) and (6), the observation equation of rotation parameters can be expressed as

(7) $$A_{r(k)}\delta_{r}=L_{r(k)},$$

where $A_{r(k)}$, $L_{r(k)}$, and $\delta_{r}$ are 3k×3 matrix, 3k×1 matrix, and 3×1 matrix, respectively, and

(8) $$A_{r(k)}=\left[\begin{array}{c} \alpha_{1}\\ \vdots \\ \alpha_{k} \end{array}\right],\;L_{r(k)}=\left[\begin{array}{c} p_{1c}^{i}-p_{1c}^{i+1}\\ \vdots \\ p_{kc}^{i}-p_{kc}^{i+1} \end{array}\right],\;\delta_{r}=\left[\begin{array}{c} {\hat{r}}_{a}\\ {\hat{r}}_{b}\\ {\hat{r}}_{c} \end{array}\right].$$

Assuming the weight matrix of $L_{r(k)}$ is $P_{r(k)}$, by using the principle of indirect adjustment [24] and $V^{T}PV=\min$, we can obtain the estimated $\delta_{r}$ for rotation parameters as

(9) $$\delta_{r}={\left(A_{r(k)}^{T}P_{r(k)}A_{r(k)}\right)}^{-1}A_{r(k)}^{T}P_{r(k)}L_{r(k)}.$$

### 2.3. Calculation of Translation Parameters

As the position of ***scanner i* + 1** in ***Scan i* + 1** is (0,0,0), with Equation (1), we can find that the position of ***scanner i* + 1** in ***Scan i*** is equal to the value of T, namely, the process of determining translation parameters is equivalent to solving the position of ***scanner i* + 1** in ***Scan i***. If the targets are regarded as GPS satellites, ***scanner i* + 1** is regarded as a GPS receiver, and the calculation method of translation parameters is equivalent to solving the position of the GPS receiver by GPS satellites, namely, spatial distance resection in GPS [22], which will not be affected by the estimated precision of rotation parameters.

If the observation value of distance between ***scanner i* + 1** and the ***j*th *target*** in ***Scan i* + 1** is $d_{j}^{i+1}$, the observation equation of translation parameters can be expressed as

(10) $$\left[\begin{array}{c} d_{1}^{i+1}\\ \vdots \\ d_{k}^{i+1} \end{array}\right]=\left[\begin{array}{c} \sqrt{{\left(x_{1}^{i}-t_{x}\right)}^{2}+{\left(y_{1}^{i}-t_{y}\right)}^{2}+{\left(z_{1}^{i}-t_{z}\right)}^{2}}\\ \vdots \\ \sqrt{{\left(x_{k}^{i}-t_{x}\right)}^{2}+{\left(y_{k}^{i}-t_{y}\right)}^{2}+{\left(z_{k}^{i}-t_{z}\right)}^{2}} \end{array}\right],$$

where $d_{j}^{i+1}=\sqrt{{\left(x_{j}^{i+1}\right)}^{2}+{\left(y_{j}^{i+1}\right)}^{2}+{\left(z_{j}^{i+1}\right)}^{2}}$ and j=1,2,⋯,k.

If the approximation values of translation parameters and corrections of translation parameters $t_{x}$, $t_{y}$, and $t_{z}$ are $t_{x,0}$, $t_{y,0}$, and $t_{z,0}$ (calculated by the method of Appendix C in [3]) and $\varepsilon_{t_{x}}$, $\varepsilon_{t_{y}}$, and $\varepsilon_{t_{z}}$, then from the linearization theorem [22,24], the linearization form of Equation (10) can be expressed as

(11) $$A_{t(k)}\delta_{t}=L_{t(k)},\;$$

where $T=T_{0}+\delta_{t}$; $A_{t(k)}$, $L_{t(k)}$, and $\delta_{t}$ are k×3 matrix, k×1 matrix, 3×1 matrix, respectively; and

(12) $$A_{t(k)}=\left[\begin{array}{c} \beta_{1}\\ \vdots \\ \beta_{k} \end{array}\right]=\left[\begin{array}{ccc} l_{x,1} & m_{y,1} & n_{z,1}\\ \vdots & \vdots & \vdots \\ l_{x,k} & m_{y,k} & n_{z,k} \end{array}\right],\;\delta_{t}=\left[\begin{array}{c} \varepsilon_{t_{x}}\\ \varepsilon_{t_{y}}\\ \varepsilon_{t_{z}} \end{array}\right],\;L_{t(k)}=\left[\begin{array}{c} d_{1,0}^{i}-d_{1}^{i+1}\\ \vdots \\ d_{k,0}^{i}-d_{k}^{i+1} \end{array}\right],\;T_{0}=\left[\begin{array}{c} t_{x,0}\\ t_{y,0}\\ t_{z,0} \end{array}\right],$$

(13) $$\beta_{j}=\left[\begin{array}{ccc} l_{x,j} & m_{y,j} & n_{z,j} \end{array}\right]=\left[\begin{array}{ccc} \frac{x_{j}^{i}-t_{x,0}}{d_{j,0}^{i}} & \frac{y_{j}^{i}-t_{y,0}}{d_{j,0}^{i}} & \frac{z_{j}^{i}-t_{z,0}}{d_{j,0}^{i}} \end{array}\right],\;j=1,2,\cdots ,k,$$

(14) $$d_{j,0}^{i}=\sqrt{{\left(x_{j}^{i}-t_{x,0}\right)}^{2}+{\left(y_{j}^{i}-t_{y,0}\right)}^{2}+{\left(z_{j}^{i}-t_{z,0}\right)}^{2}}.$$

Assuming the weight matrix of $L_{t(k)}$ is $P_{t(k)}$, by using the principle of indirect adjustment [24], we can obtain the estimated $\delta_{t}$ for translation parameters as

(15) $$\delta_{t}={\left(A_{t(k)}^{T}P_{t(k)}A_{t(k)}\right)}^{-1}A_{t(k)}^{T}P_{t(k)}L_{t(k)}.$$

### 2.4. Quantitative Evaluation Model of TGD

With Equations (9) and (15), based on the theorem of error propagation [24], the covariance $D_{\delta_{r}\delta_{r}}$ and $D_{\delta_{t}\delta_{t}}$ of the rotation parameters $\delta_{r}$ and the translation parameter corrections $\delta_{t}$ can be obtained as

(16) $$\left\{ \begin{array}{l} D_{\delta_{r}\delta_{r}}={\left(A_{r(k)}^{T}P_{r(k)}A_{r(k)}\right)}^{-1}\sigma_{0}^{2}\\ D_{\delta_{t}\delta_{t}}={\left(A_{t(k)}^{T}P_{t(k)}A_{t(k)}\right)}^{-1}\sigma_{0}^{2} \end{array}\right.,$$

where $D_{\delta_{r}\delta_{r}}$ and $D_{\delta_{t}\delta_{t}}$ are 3×3 matrices, and $\sigma_{0}$ is the unit weight variance, usually determined in the initial processing before registration.

As the trace of a real-symmetric matrix is equal to the trace of its corresponding diagonal matrix and the parameters’ variance-covariance matrix is a real-symmetric matrix, we usually use the trace of the parameters’ variance-covariance matrix in the precision evaluation of parameters, such as point precision evaluation. For this reason, we assume that the variances of rotation parameters ${\hat{r}}_{a}$, ${\hat{r}}_{b}$, and ${\hat{r}}_{c}$ and the translation parameters’ corrections $\varepsilon_{t_{x}}$, $\varepsilon_{t_{y}}$, and $\varepsilon_{t_{z}}$ are $\sigma_{a}$, $\sigma_{b}$, $\sigma_{c}$, $\sigma_{t_{x}}$, $\sigma_{t_{y}}$, and $\sigma_{t_{z}}$, respectively. With Equation (16), the registration precision (namely, the variances of parameters $\delta_{r}$ and $\delta_{t}$) can be obtained as

(17) $$\sqrt{\sigma_{a}^{2}+\sigma_{b}^{2}+\sigma_{c}^{2}}=tr(\sqrt{D_{\delta_{r}\delta_{r}}})=tr(\sqrt{{\left(A_{r(k)}^{T}P_{r(k)}A_{r(k)}\right)}^{-1}})\sigma_{0},$$

(18) $$\sqrt{\sigma_{t_{x}}^{2}+\sigma_{t_{y}}^{2}+\sigma_{t_{z}}^{2}}=tr(\sqrt{D_{\delta_{t}\delta_{t}}})=tr(\sqrt{{\left(A_{t(k)}^{T}P_{t(k)}A_{t(k)}\right)}^{-1}})\sigma_{0},$$

where *tr*(.) is the trace of the matrix. 

In GPS positioning, the impact of the satellites’ geometry distribution on the positioning quality is evaluated by the dilution of precision (DOP) values [25,26,27]. Similarly, we can also build a quantitative evaluation model of the impact of TGD on the registration precision, that is, the rotation dilution of precision (rDOP) and the translation dilution of precision (tDOP), namely

(19) $$rDOP=\sqrt{tr(G_{k}^{-1})},$$

(20) $$tDOP=\sqrt{tr(H_{k}^{-1})},$$

where $G_{k}=A_{r(k)}^{T}P_{r(k)}A_{r(k)}$ and $H_{k}=A_{t(k)}^{T}P_{t(k)}A_{t(k)}$.

With Equations (17)–(20), we can find that the registration precision of rotation parameters and translation parameters are

(21) $$\sqrt{\sigma_{a}^{2}+\sigma_{b}^{2}+\sigma_{c}^{2}}=rDOP\cdot \sigma_{0},$$

(22) $$\sqrt{\sigma_{t_{x}}^{2}+\sigma_{t_{y}}^{2}+\sigma_{t_{z}}^{2}}=tDOP\cdot \sigma_{0}.$$

From the above evaluation model of TGD, we can find that

1. The values of rDOP and tDOP represent the amplification of the unit weight variance, which means the lower the values of rDOP and tDOP, the higher the solution precisions of the rotation parameters and the translation parameters;
2. The values of rDOP calculated by Equation (19) are related to the coefficient matrix $A_{r(k)}$ and the weight matrix $P_{r(k)}$ of $L_{r(k)}$, among which $A_{r(k)}$ is related to TGD (the coordinates of targets $p_{jc}^{i}$) and the rotation matrix R. Namely, when R is fixed, the better the quality of TGD, the lower the values of rDOP;
3. The values of tDOP calculated by Equation (20) are related to the coefficient matrix $A_{t(k)}$; and the weight matrix $P_{t(k)}$ of $L_{t(k)}$, among which $A_{t(k)}$ is related to TGD (the coordinates of targets $p_{j}^{i}$) and the position of ***scanner i* + 1** in ***Scan i***. Namely, the better the quality of TGD and the position of ***scanner i* + 1**, the lower the values of tDOP;
4. The calculation formula of tDOP is identical to the calculation formula of DOP in GPS, so the tDOP can be used to evaluate the quality of the received GPS satellites’ distribution. Namely, the rDOP and tDOP model is a unified evaluation model of the targets’ and GNSS satellites’ geometric distribution.

### 2.5. Equationuationual Weight Model of rDOP and tDOP

In constituting the DOP model for evaluating the impact of the selected GNSS satellite geometry [25,26,27], we usually assume that the weight matrix is an identity matrix. Additionally, in all the empirical experiments and simulations of the TGD impact on the registration precision [2,12,14,15,16,17,18,19,20], we assume that the weight matrix is an identity matrix. For these reasons and for convenience of the following analysis on the nature of the rDOP and tDOP model, we assume that $P_{r(k)}$ and $P_{t(k)}$ are equal to identity matrix I, and use the equal weight least squares method to compute the registration parameters in Equations (9) and (15). With Equations (6)–(8) and Equations (12)–(14), we then get

(23) $$G_{k}=A_{r(k)}^{T}A_{r(k)}=\sum_{j=1}^{k}\left[\begin{array}{ccc} \tau_{j}^{2}-\tau_{x,j}^{2} & -\tau_{x,j}\tau_{y,j} & -\tau_{x,j}\tau_{z,j}\\ -\tau_{x,j}\tau_{y,j} & \tau_{j}^{2}-\tau_{y,j}^{2} & -\tau_{y,j}\tau_{z,j}\\ -\tau_{x,j}\tau_{z,j} & -\tau_{y,j}\tau_{z,j} & \tau_{j}^{2}-\tau_{z,j}^{2} \end{array}\right],$$

(24) $$H_{k}=A_{t(k)}^{T}A_{t(k)}=\sum_{j=1}^{k}\left[\begin{array}{ccc} l_{x,j}^{2} & -l_{x,j}m_{y,j} & -l_{x,j}n_{z,j}\\ -l_{x,j}m_{y,j} & l_{y,j}^{2} & -m_{y,j}n_{z,j}\\ -l_{x,j}n_{z,j} & -m_{y,j}n_{z,j} & l_{z,j}^{2} \end{array}\right],$$

where $\tau_{j}^{2}=\tau_{x,j}^{2}+\tau_{y,j}^{2}+\tau_{z,j}^{2}={\left(x_{jc}^{i}+x_{jc}^{i+1}\right)}^{2}+{\left(y_{jc}^{i}+y_{jc}^{i+1}\right)}^{2}+{\left(z_{jc}^{i}+z_{jc}^{i+1}\right)}^{2}$.

Through the simulation method similar to [13], we can find that the relationship curve between rDOP and the rotation angle of the rotation matrix R under different TGDs is different and increases monotonically, and the relationship curves corresponding to different TGDs do not intersect. Therefore, we can compare the rDOP values of different TGDs under the rotation angle of the rotation matrix R by the values of rDOP under the rotation angle of the unit matrix $I_{3}$ (namely, the conclusions of rDOP under arbitrary rotation matrix R are equivalent to the conclusions under $R=I_{3}$). Then, we can evaluate the quality of TGD by only the values of rDOP under $R=I_{3}$, and Equation (23) can be written as

(25) $$G_{k}=4\sum_{j=1}^{k}\left[\begin{array}{ccc} {\left(y_{jc}^{i}\right)}^{2}+{\left(z_{jc}^{i}\right)}^{2} & -x_{jc}^{i}y_{jc}^{i} & -x_{jc}^{i}z_{jc}^{i}\\ -x_{jc}^{i}y_{jc}^{i} & {\left(x_{jc}^{i}\right)}^{2}+{\left(z_{jc}^{i}\right)}^{2} & -y_{jc}^{i}z_{jc}^{i}\\ -x_{jc}^{i}z_{jc}^{i} & -y_{jc}^{i}z_{jc}^{i} & {\left(x_{jc}^{i}\right)}^{2}+{\left(y_{jc}^{i}\right)}^{2} \end{array}\right].$$

### 2.6. Model Application Scheme

In order to use our proposed evaluation model, here, we give the implementation procedures for three kinds of situations: (1) the position of all targets are known, so we need to determine the optimum setting position of ***scanner i* + 1** in ***Scan i,*** namely, the best position of ***scanner i* + 1**; (2) the position of ***scanner i* + 1** is known, so we need to determine the optimum setting positions of targets, namely, the best TGD; and (3) we need to determine both the optimum positions of targets and the ***scanner i* + 1** in ***Scan i,*** namely, the best TGD and the best position of ***scanner i* + 1**.

#### 2.6.1. The Best Position of ***Scanner i* + 1**

The best position of ***Scanner ii* + 1** can be identified as follows:

(1) Selecting the possible place $\left\{o_{1}^{i},\cdots ,o_{m}^{i}\right\}$ of ***scanner i* + 1** in ***Scan i***;
(2) Obtaining the coordinates of all targets in ***Scan i***;
(3) Using Equations (11), (12), (20) and (24) to calculate the values of tDOP under different possible places of ***scanner i* + 1**;
(4) When the value of tDOP is the minimum, the corresponding place is the best position of ***scanner i* + 1**.

#### 2.6.2. The Best TGD

The best TGD can be identified as follows:

(1) Selecting the possible place $\left\{p_{1}^{i},\cdots ,p_{m}^{i}\right\}$ of targets in ***Scan i***;
(2) Obtaining the coordinates of ***scanner i* + 1** in ***Scan i***;
(3) Setting the number *k* of targets;
(4) Choosing *k* places from $\left\{p_{1}^{i},\cdots ,p_{m}^{i}\right\}$, and using Equations (6), (20) and (25) to calculate the values of rDOP;
(5) When the value of rDOP is the minimum, the corresponding places are the optimum positions of targets, namely, the best TGD.

#### 2.6.3. The Best TGD and the Best Position of ***Scanner i* + 1**

From Section 2.4, it can be known that the rDOP model is mainly related to TGD, and the tDOP model is related to TGD and the position of ***scanner i* + 1**′s origin, which is relative to the selected TGD. Therefore, we firstly determined the best TGD by rDOP, and then determined the best position of ***scanner i* + 1** by tDOP. The implementation procedures are as follows:

(1) Selecting the possible place $\left\{p_{1}^{i},\cdots ,p_{m}^{i}\right\}$ of targets and the possible place $\left\{o_{1}^{i},\cdots ,o_{m}^{i}\right\}$ of *scanner i* **+ 1** in *Scan i*;
(2) Setting the number *k* of targets;
(3) Similar to the above, calculating the values of rDOP by *k* different possible places of targets, and selecting the best TGD where the value of rDOP is the minimum;
(4) Similar to the above, calculating the values of tDOP under different possible places of ***scanner i* + 1,** and selecting the best position of ***scanner i* + 1** where the value of tDOP is the minimum.

## 3. Theoretical Analysis

We first theoretically analyzed the existence conditions of rDOP and tDOP. We then theoretically analyzed the relationship of “rDOP and tDOP” and the number of targets. Finally, we analyzed the bounds of rDOP and tDOP.

### 3.1. The Existence Conditions of tDOP

The existence condition of tDOP is that the matrix $H_{k}$ is invertible, which is equal to $\left|H_{k}\right|\neq 0$, namely, the rank of $A_{t(k)}$ is 3.

If all targets and ***scanner i* + 1** are on the same plane, and assuming the plane equation is $c_{1}x+c_{2}y+c_{3}z+c_{4}=0$, with Equation (13), we can get

(26) $$c_{1}\frac{x_{j}^{i}}{d_{j,0}^{i}}+c_{2}\frac{y_{j}^{i}}{d_{j,0}^{i}}+c_{3}\frac{z_{j}^{i}}{d_{j,0}^{i}}+c_{4}=0,$$

(27) $$c_{1}\frac{t_{x,0}}{d_{j,0}^{i}}+c_{2}\frac{t_{y,0}}{d_{j,0}^{i}}+c_{3}\frac{t_{z,0}}{d_{j,0}^{i}}+c_{4}=0.$$

Then, Equation (26) minus Equation (27) is

(28) $$c_{1}\frac{x_{j}^{i}-t_{x,0}}{d_{j,0}^{i}}+c_{2}\frac{y_{j}^{i}-t_{y,0}}{d_{j,0}^{i}}+c_{3}\frac{z_{j}^{i}-t_{z,0}}{d_{j,0}^{i}}=0.$$

With Equations (12), (13) and (28), we can get

(29) $$A_{t(k)}\left[\begin{array}{c} c_{1}\\ c_{2}\\ c_{3} \end{array}\right]=0,$$

where the equation has a non-zero solution if and only if the rank of $A_{t(k)}$ is less than 3.

Therefore, the existence condition of tDOP is that all targets and ***scanner i* + 1** are not on the same plane, which theoretically proves the experience that “**all targets and *scanner i* + 1 should not** **lie on the same plane**” [2,12].

### 3.2. The Existence Conditions of rDOP

The existence condition of rDOP is that the matrix $G_{k}$ is invertible, which is equal to $\left|G_{k}\right|\neq 0$.

With Equation (23), using the property of matrix inversion, we can get

(30) $${\left(G_{k}\right)}^{-1}=\frac{1}{\left|G_{k}\right|}\left[\begin{array}{ccc} g_{11} & g_{12} & g_{13}\\ g_{21} & g_{22} & g_{23}\\ g_{31} & g_{32} & g_{33} \end{array}\right],$$

where

(31) $$\begin{array}{ll} \left|G_{k}\right| & =\sum_{j=1}^{k}\tau_{j}^{2}\left[\sum_{j=1}^{k}\tau_{x,j}^{2}\sum_{j=1}^{k}\tau_{y,j}^{2}+\sum_{j=1}^{k}\tau_{x,j}^{2}\sum_{j=1}^{k}\tau_{z,j}^{2}+\sum_{j=1}^{k}\tau_{y,j}^{2}\sum_{j=1}^{k}\tau_{z,j}^{2}\right]\\ & -\sum_{j=1}^{k}\tau_{j}^{2}\left[{\left(\sum_{j=1}^{k}\tau_{x,j}\tau_{y,j}\right)}^{2}+{\left(\sum_{j=1}^{k}\tau_{x,j}\tau_{z,j}\right)}^{2}+{\left(\sum_{j=1}^{k}\tau_{y,j}\tau_{z,j}\right)}^{2}\right]\\ & +\sum_{j=1}^{k}\tau_{x,j}^{2}{\left(\sum_{j=1}^{k}\tau_{y,j}\tau_{z,j}\right)}^{2}+\sum_{j=1}^{k}\tau_{y,j}^{2}{\left(\sum_{j=1}^{k}\tau_{x,j}\tau_{z,j}\right)}^{2}+\sum_{j=1}^{k}\tau_{z,j}^{2}{\left(\sum_{j=1}^{k}\tau_{x,j}\tau_{y,j}\right)}^{2}\\ & -\sum_{j=1}^{k}\tau_{x,j}^{2}\sum_{j=1}^{k}\tau_{y,j}^{2}\sum_{j=1}^{k}\tau_{z,j}^{2}-2\sum_{j=1}^{k}\tau_{x,j}\tau_{y,j}\sum_{j=1}^{k}\tau_{x,j}\tau_{z,j}\sum_{j=1}^{k}\tau_{y,j}\tau_{z,j} \end{array}$$

(32) $$g_{11}=\sum_{j=1}^{k}\tau_{j}^{2}\sum_{j=1}^{k}\tau_{x,j}^{2}+\sum_{j=1}^{k}\tau_{y,j}^{2}\sum_{j=1}^{k}\tau_{z,j}^{2}-{\left(\sum_{j=1}^{k}\tau_{y,j}\tau_{z,j}\right)}^{2},$$

(33) $$g_{22}=\sum_{j=1}^{k}\tau_{j}^{2}\sum_{j=1}^{k}\tau_{y,j}^{2}+\sum_{j=1}^{k}\tau_{x,j}^{2}\sum_{j=1}^{k}\tau_{z,j}^{2}-{\left(\sum_{j=1}^{k}\tau_{x,j}\tau_{z,j}\right)}^{2},$$

(34) $$g_{33}=\sum_{j=1}^{k}\tau_{j}^{2}\sum_{j=1}^{k}\tau_{z,j}^{2}+\sum_{j=1}^{k}\tau_{x,j}^{2}\sum_{j=1}^{k}\tau_{y,j}^{2}-{\left(\sum_{j=1}^{k}\tau_{x,j}\tau_{y,j}\right)}^{2}.$$

From the inequality $\sum_{i=1}^{k}x_{i}^{2}\sum_{i=1}^{k}y_{i}^{2}\geq {\left(\sum_{i=1}^{k}x_{i}y_{i}\right)}^{2}$, we know

(35) $$g_{11}\geq 0,\;g_{22}\geq 0,\;g_{33}\geq 0,$$

where equality is achieved if and only if $\tau_{x,j}=\tau_{y,j}=\tau_{z,j}=0$, which is equivalent to the situation that any centralized targets $p_{jc}^{i+1}$ in ***Scan i* + 1** satisfy $(R+I_{3})p_{jc}^{i+1}=0$, namely, the following three situations are true:

1. All targets are in the plane $x^{i}oy^{i}$ of ***Scan i***’s coordinate system, and $R=\left[\begin{array}{ccc} -1 & & \\ & -1 & \\ & & 1 \end{array}\right]$;
2. All targets are in the plane $x^{i}oz^{i}$ of ***Scan i***’s coordinate system, and $R=\left[\begin{array}{ccc} -1 & & \\ & 1 & \\ & & -1 \end{array}\right]$;
3. All targets are in the plane $y^{i}oz^{i}$ of ***Scan i***’s coordinate system, and $R=\left[\begin{array}{ccc} 1 & & \\ & -1 & \\ & & -1 \end{array}\right]$.

From the inequality $\sum_{i=1}^{k}x_{i}^{2}\sum_{i=1}^{k}y_{i}^{2}\geq {\left(\sum_{i=1}^{k}x_{i}y_{i}\right)}^{2}$, we also know

(36) $$\sum_{j=1}^{k}\tau_{x,j}\tau_{y,j}\sum_{j=1}^{k}\tau_{x,j}\tau_{z,j}\sum_{j=1}^{k}\tau_{y,j}\tau_{z,j}\leq \sum_{j=1}^{k}\tau_{x,j}^{2}\sum_{j=1}^{k}\tau_{y,j}^{2}\sum_{j=1}^{k}\tau_{z,j}^{2}.$$

Combined with Equations (31) and (36), we can get

(37) $$\begin{array}{cc} \left|G_{k}\right| & \geq \sum_{j=1}^{k}\tau_{x,j}^{2}\left[\sum_{j=1}^{k}\tau_{x,j}^{2}\sum_{j=1}^{k}\tau_{y,j}^{2}+\sum_{j=1}^{k}\tau_{x,j}^{2}\sum_{j=1}^{k}\tau_{z,j}^{2}-{\left(\sum_{j=1}^{k}\tau_{x,j}\tau_{y,j}\right)}^{2}-{\left(\sum_{j=1}^{k}\tau_{x,j}\tau_{z,j}\right)}^{2}\right]\\ & +\sum_{j=1}^{k}\tau_{y,j}^{2}\left[\sum_{j=1}^{k}\tau_{x,j}^{2}\sum_{j=1}^{k}\tau_{y,j}^{2}+\sum_{j=1}^{k}\tau_{y,j}^{2}\sum_{j=1}^{k}\tau_{z,j}^{2}-{\left(\sum_{j=1}^{k}\tau_{x,j}\tau_{y,j}\right)}^{2}-{\left(\sum_{j=1}^{k}\tau_{y,j}\tau_{z,j}\right)}^{2}\right]\\ & +\sum_{j=1}^{k}\tau_{z,j}^{2}\left[\sum_{j=1}^{k}\tau_{x,j}^{2}\sum_{j=1}^{k}\tau_{z,j}^{2}+\sum_{j=1}^{k}\tau_{y,j}^{2}\sum_{j=1}^{k}\tau_{z,j}^{2}-{\left(\sum_{j=1}^{k}\tau_{x,j}\tau_{z,j}\right)}^{2}-{\left(\sum_{j=1}^{k}\tau_{y,j}\tau_{z,j}\right)}^{2}\right] \end{array}$$

where equality is achieved if and only if $\tau_{x,j}=\gamma_{xy}\tau_{y,j}=\gamma_{xz}\tau_{z,j}$, which is equivalent to the situation that all targets lay on the same line. 

From Equation (37), we know that “the value of $\left|G_{k}\right|$ is smaller when the TGD is closer to a straight line, the value of rDOP is larger, and the precision of the rotation parameters solution is worse, while if all targets lay on the same line, $\left|G_{k}\right|=0$”. Therefore, the existence condition of rDOP is that all targets are not on the same line, or the above three situations are not satisfied, which theoretically proves the experience that “**the TLS targets should be distributed evenly over the overlapped space and should not lie on the same line or be close”** [2,17].

### 3.3. The Relationship Between tDOP and the Number of Targets

If more targets are considered (over *k*), the $A_{t(k)}$ can be successively augmented by adding row vectors. For example, if there are k+1 targets considered, then

(38) $$A_{t(k+1)}=\left[\begin{array}{c} A_{t(k)}\\ \beta_{k+1} \end{array}\right],$$

in which we assume that $A_{t(k)}$ is nonsingular and $\beta_{k+1}$ is a nonzero vector, and

(39) $$\beta_{k+1}=\left[\begin{array}{ccc} l_{x,k+1} & m_{y,k+1} & n_{z,k+1} \end{array}\right]=\left[\begin{array}{ccc} \frac{x_{k+1}^{i}-t_{x,0}}{d_{k+1,0}^{i}} & \frac{y_{k+1}^{i}-t_{y,0}}{d_{k+1,0}^{i}} & \frac{z_{k+1}^{i}-t_{z,0}}{d_{k+1,0}^{i}} \end{array}\right].$$

Yarlagadda et al. [27] proved that increasing the number of satellites will reduce the DOP in GPS applications. Here, we take the same derivation method described by Yarlagadda et al. [27] to prove its effectiveness in tDOP. With Equation (38), we can get

(40) $$A_{t(k+1)}^{T}A_{t(k+1)}=A_{t(k)}^{T}A_{t(k)}+\beta_{k+1}^{T}\beta_{k+1}.$$

By using the inversion formulas of matrix [2], we can get

(41) $$H_{k+1}^{-1}={\left(H_{k}+\beta_{k+1}^{T}\beta_{k+1}\right)}^{-1}=H_{k}^{-1}-\frac{H_{k}^{-1}\beta_{k+1}^{T}\beta_{k+1}H_{k}^{-1}}{1+\beta_{k+1}H_{k}^{-1}\beta_{k+1}^{T}}.$$

It is clear that $H_{k}^{-1}$ is a positive definite symmetric matrix, which can be denoted as $H_{k}^{-1}=U^{T}U$, and U is the upper triangular matrix. Let $\eta =\beta_{k+1}H_{k}^{-1}$ and $\mu =\beta_{k+1}U^{T}$, where η and μ are 1×3 real-valued vectors, $\eta \eta^{T}\geq 0$, and $\mu \mu^{T}\geq 0$. Through using the property of the matrix trace, we can write

(42) $$tr(H_{k+1}^{-1})=tr(H_{k}^{-1})-\frac{\eta \eta^{T}}{1+\mu \mu^{T}}.$$

Then, we can get $tr(H_{k+1}^{-1})<tr(H_{k}^{-1})$, which means that increasing the number of targets will reduce the value of tDOP and improve the registration precision, which theoretically proves the experience that “**the more targets, the higher the registration precision**” [2,17,18,19,20,27].

### 3.4. The Relationship Between rDOP and the Number of Targets

Similar to tDOP, if more targets (over k) are considered, matrix $A_{r(k)}$ can also be successively augmented by adding row vectors. For example, if there are k+1 targets considered, then

(43) $$\Omega_{1}=\left[\begin{array}{c} A_{r(k)}\\ \alpha_{k+1(1)} \end{array}\right],\;\Omega_{2}=\left[\begin{array}{c} \Omega_{1}\\ \alpha_{k+1(2)} \end{array}\right],\;A_{r(k+1)}=\left[\begin{array}{c} A_{r(k)}\\ \begin{array}{c} \alpha_{k+1(1)}\\ \alpha_{k+1(2)}\\ \alpha_{k+1(3)} \end{array} \end{array}\right],$$

where

(44) $$\left\{ \begin{array}{l} \alpha_{k+1(1)}=\left[\begin{array}{ccc} 0 & -\tau_{z,k+1} & \tau_{y,k+1} \end{array}\right]\\ \alpha_{k+1(2)}=\left[\begin{array}{ccc} \tau_{z,k+1} & 0 & -\tau_{x,k+1} \end{array}\right]\\ \alpha_{k+1(3)}=\left[\begin{array}{ccc} -\tau_{y,k+1} & \tau_{x,k+1} & 0 \end{array}\right] \end{array}\right.\;\mathrm{and}\;\left\{ \begin{array}{l} \tau_{x,k+1}=x_{k+1,c}^{i}+x_{k+1,c}^{i+1}\\ \tau_{y,k+1}=y_{k+1,c}^{i}+y_{k+1,c}^{i+1}\\ \tau_{z,k+1}=z_{k+1,c}^{i}+z_{k+1,c}^{i+1} \end{array}\right..$$

Assume that $A_{r(k)}$ is nonsingular and $\alpha_{k+1(1)}$, $\alpha_{k+1(2)}$, and $\alpha_{k+1(3)}$ are nonzero vectors. By taking the same derivation method of Equations (38)–(42), we can get

(45) $$tr(G_{k+1}^{-1})<tr({\left(\Omega_{2}^{T}\Omega_{2}\right)}^{-1})<tr({\left(\Omega_{1}^{T}\Omega_{1}\right)}^{-1})<tr(G_{k}^{-1}).$$

Therefore, increasing the number of targets will reduce the value of rDOP and improve the registration precision, which also theoretically proves the experience in Section 3.3.

### 3.5. tDOP Bounds

To find the optimum position of ***scanner i* + 1**, we need to analyze the bounds of tDOP, namely, the minimum of tDOP.

Denoting the three eigenvalues of $H_{k}$ are $\lambda_{t,1}$, $\lambda_{t,2}$, and $\lambda_{t,3}$, and using the property of matrix eigenvalues, we know that $\frac{1}{\lambda_{t,1}}$, $\frac{1}{\lambda_{t,2}}$, and $\frac{1}{\lambda_{t,3}}$ are the three eigenvalues of $H_{k}^{-1}$. Then, the tDOP can be rewritten as

(46) $$tDOP=\sqrt{tr(H_{k}^{-1})}=\sqrt{\frac{1}{\lambda_{t,1}}+\frac{1}{\lambda_{t,2}}+\frac{1}{\lambda_{t,3}}}.$$

With Equations (13) and (24), we can get

(47) $$\lambda_{t,1}+\lambda_{t,2}+\lambda_{t,3}=tr(H_{k})=\sum_{j=1}^{k}\left(l_{x,j}^{2}+m_{y,j}^{2}+n_{z,j}^{2}\right)=k.$$

Let $f=\sqrt{\frac{1}{\lambda_{t,1}}+\frac{1}{\lambda_{t,2}}+\frac{1}{\lambda_{t,3}}}+\mu (\lambda_{t,1}+\lambda_{t,2}+\lambda_{t,3}-k)$, and using the method of Lagrange multipliers as described in [15], we can get

(48) $$tDOP=\sqrt{tr(H_{k}^{-1})}\geq \frac{3}{\sqrt{k}}.$$

The equality of Equation (48) is achieved if and only if $\lambda_{t,1}=\lambda_{t,2}=\lambda_{t,3}=\frac{k}{3}$, which is equivalent to $\sum_{j=1}^{k}l_{x,j}^{2}=\sum_{j=1}^{k}m_{y,j}^{2}=\sum_{j=1}^{k}n_{z,j}^{2}=\frac{k}{3}$; that is, “the polyhedron $p_{1}^{i}p_{2}^{i}\cdots p_{k}^{i}$ is regular” and “the position of ***scanner i* +1** is the barycenter of all targets”. This characteristic theoretically proves the experience that “**the best setting position of *scanner i* + 1 is the barycenter of all targets**” [12].

Furthermore, from Equation (48), it can be seen that the minimum value of tDOP is $\frac{3}{\sqrt{k}}$, which shows that increasing the number of targets will reduce the minimum value of tDOP.

### 3.6. rDOP Bounds

To find the optimum TGD, we need to analyze the bounds of rDOP, namely, the minimum of rDOP.

Denoting the three eigenvalues of $G_{k}$ are $\lambda_{r,1}$, $\lambda_{r,2}$, and $\lambda_{r,3}$, and using the property of matrix eigenvalues, we know that $\frac{1}{\lambda_{r,1}}$, $\frac{1}{\lambda_{r,2}}$, and $\frac{1}{\lambda_{r,3}}$ are the three eigenvalues of $G_{k}^{-1}$, so with Equations (19) and (23), we can get

(49) $$\lambda_{r,1}+\lambda_{r,2}+\lambda_{r,3}=tr(G_{k})=2\sum_{j=1}^{k}\tau_{j}^{2},$$

(50) $$rDOP=\sqrt{tr(G_{k}^{-1})}=\sqrt{\sum_{j=1}^{3}\frac{1}{\lambda_{r,j}}}\geq \frac{3}{\sqrt{2\sum_{j=1}^{k}\tau_{j}^{2}}},$$

where equality is achieved if and only if $\lambda_{r,1}=\lambda_{r,2}=\lambda_{r,3}=\frac{2}{3}\sum_{j=1}^{k}\tau_{j}^{2}$, which is equivalent to $\sum_{j=1}^{k}\tau_{x,j}^{2}=\sum_{j=1}^{k}\tau_{y,j}^{2}=\sum_{j=1}^{k}\tau_{z,j}^{2}$.

Denoting the distance between the barycenter of all targets and the ***jth* target** in ***Scan i*** or ***Scan i* + 1** are $d_{jc}^{i}$ or $d_{jc}^{i+1}$, respectively, so

(51) $${\left(d_{jc}^{i}\right)}^{2}={\left(p_{jc}^{i}\right)}^{T}p_{jc}^{i}={\left(Rp_{jc}^{i+1}\right)}^{T}Rp_{jc}^{i+1}={\left(p_{jc}^{i+1}\right)}^{T}p_{jc}^{i+1}={\left(d_{jc}^{i+1}\right)}^{2}.$$

Based on the inequality equation ${\left(x+y\right)}^{2}\leq 2(x^{2}+y^{2})$, we know

(52) $$\sum_{j=1}^{k}\tau_{j}^{2}\leq 2\sum_{j=1}^{k}\left({\left(d_{jc}^{i}\right)}^{2}+{\left(d_{jc}^{i+1}\right)}^{2}\right)=4\sum_{j=1}^{k}{\left(d_{jc}^{i}\right)}^{2},$$

where equality is achieved if and only if $x_{jc}^{i}=x_{jc}^{i+1}$, $y_{jc}^{i}=y_{jc}^{i+1}$, and $z_{jc}^{i}=z_{jc}^{i+1}$, which is equivalent to the rotation matrix R being the identity matrix $I_{3}$.

With Equations (50) and (52), we can then get

(53) $$rDOP=\sqrt{tr(G_{k}^{-1})}\geq \frac{3}{\sqrt{8\sum_{j=1}^{k}{\left(d_{jc}^{i}\right)}^{2}}},$$

where equality of Equation (53) is achieved if and only if $R=I_{3}$ and $\sum_{j=1}^{k}x_{jc}^{2}=\sum_{j=1}^{k}y_{jc}^{2}=\sum_{j=1}^{k}z_{jc}^{2}$. 

Equation (53) indicates that the minimum value of rDOP is inversely proportional to the sum of the distances $d_{jc}^{i}$ from the targets to the barycenter of all targets. Therefore, without considering the precision of target extraction, the more targets disperse, the smaller the minimum rDOP and the higher the registration precision. This theoretically proves the experience that “**the more dispersive the targets, the higher the registration precision**” [3,17,18].

## 4. Experimental Verification

In practical applications, we might calculate all the registration parameters together (while the above model is deduced by separating translation and rotation parameters), so we need to analyze the applicability of the quantitative evaluation model of the TGD without separating translation and rotation. For these reasons, we first introduce the method of calculating the registration precision without separating translation and rotation, then design two experiments to verify the quantitative evaluation model of the TGD, and finally analyze the experiments’ results.

### 4.1. Calculation Method of Registration Precision

The precision of target-based registration can be evaluated by the root mean square errors of rotation parameters ($RMSE_{r}$) and translation parameters ($RMSE_{t}$). The specific experimental processes are as follows:

Step 1: Input the coordinate true values of target ${\tilde{p}}_{j}^{i}(j=1,2,\cdots ,k)$ and the true values of transformation parameters ${\tilde{r}}_{a}$, ${\tilde{r}}_{b}$, ${\tilde{r}}_{c}$, ${\tilde{t}}_{x}$, ${\tilde{t}}_{y}$, and ${\tilde{t}}_{z}$;

Step 2: Calculate the target coordinates in ***Scan i* + 1**:

(54) $${\tilde{p}}_{j}^{i+1}=\tilde{R}({\tilde{p}}_{j}^{i}-T),\;j=1,2,\cdots ,k,$$

where

(55) $$\tilde{R}=\frac{1}{1+{\tilde{r}}_{a}^{2}+{\tilde{r}}_{b}^{2}+{\tilde{r}}_{c}^{2}}\left[\begin{array}{ccc} 1+{\tilde{r}}_{a}^{2}-{\tilde{r}}_{b}^{2}-{\tilde{r}}_{c}^{2} & 2({\tilde{r}}_{c}+{\tilde{r}}_{a}{\tilde{r}}_{b}) & 2({\tilde{r}}_{a}{\tilde{r}}_{c}-{\tilde{r}}_{b})\\ 2({\tilde{r}}_{a}{\tilde{r}}_{b}-{\tilde{r}}_{c}) & 1-{\tilde{r}}_{a}^{2}+{\tilde{r}}_{b}^{2}-{\tilde{r}}_{c}^{2} & 2({\tilde{r}}_{a}+{\tilde{r}}_{b}{\tilde{r}}_{c})\\ 2({\tilde{r}}_{b}+{\tilde{r}}_{a}{\tilde{r}}_{c}) & 2({\tilde{r}}_{b}{\tilde{r}}_{c}-{\tilde{r}}_{a}) & 1-{\tilde{r}}_{a}^{2}-{\tilde{r}}_{b}^{2}+{\tilde{r}}_{c}^{2} \end{array}\right],$$

(56) $$\tilde{T}=\left[\begin{array}{c} {\tilde{t}}_{x}\\ {\tilde{t}}_{y}\\ {\tilde{t}}_{z} \end{array}\right];$$

Step 3: Assume $j_{0}=1$ and the unit weight variance $\sigma_{0}=5mm$;

Step 4: If $j_{0}>1000$, go to Step 10; if not, continue;

Step 5: Add random noise to the coordinates:

(57) $$p_{j}^{i}={\tilde{p}}_{j}^{i}+normrnd(0,\sigma_{0},3,1),\;j=1,2,\cdots ,k,$$

(58) $$p_{j}^{i+1}={\tilde{p}}_{j}^{i+1}+normrnd(0,\sigma_{0},3,1),\;j=1,2,\cdots ,k,$$

where $normrnd(0,\sigma_{0},3,1)$ returns a 3×1 array of random numbers chosen from a normal distribution with the mean and standard deviation as 0 and $\sigma_{0}$;

Step 6: Calculate the approximate values of rotation parameters $r_{a,0}$, $r_{b,0}$, and $r_{c,0}$ by Equations (8) and (9);

Step 7: Calculate the approximate values of translation parameters by Equation (1):

(59) $$T_{0}=\left[\begin{array}{c} t_{x,0}\\ t_{y,0}\\ t_{z,0} \end{array}\right]=\frac{1}{k}(\sum_{j=1}^{k}p_{j}^{i}-R\sum_{j=1}^{k}p_{j}^{i+1}),$$

where

(60) $$R_{0}=\frac{1}{1+r_{a,0}^{2}+r_{b,0}^{2}+r_{c,0}^{2}}\left[\begin{array}{ccc} 1+r_{a,0}^{2}-r_{b,0}^{2}-r_{c,0}^{2} & 2(r_{c,0}+r_{a,0}r_{b,0}) & 2(r_{a,0}r_{c,0}-r_{b,0})\\ 2(r_{a,0}r_{b,0}-r_{c,0}) & 1-r_{a,0}^{2}+r_{b,0}^{2}-r_{c,0}^{2} & 2(r_{a,0}+r_{b,0}r_{c,0})\\ 2(r_{b,0}+r_{a,0}r_{c,0}) & 2(r_{b,0}r_{c,0}-r_{a,0}) & 1-r_{a,0}^{2}-r_{b,0}^{2}+r_{c,0}^{2} \end{array}\right];$$

Step 8: Calculate the estimated transformation parameters [3]:

(61) $$\left[\begin{array}{c} {\hat{r}}_{a,j_{0}}\\ {\hat{r}}_{b,j_{0}}\\ {\hat{r}}_{c,j_{0}}\\ {\hat{t}}_{x,j_{0}}\\ {\hat{t}}_{y,j_{0}}\\ {\hat{t}}_{z,j_{0}} \end{array}\right]=\left[\begin{array}{c} r_{a,0}\\ r_{b,0}\\ r_{c,0}\\ t_{x,0}\\ t_{y,0}\\ t_{z,0} \end{array}\right]+{\left(B^{T}B\right)}^{-1}B^{T}L,$$

where

(62) $$B=\left[\begin{array}{c} B_{1}\\ \vdots \\ B_{k} \end{array}\right],\;B_{j}=\left[\begin{array}{cccc} \frac{\partial R}{\partial a}p_{j}^{i+1} & \frac{\partial R}{\partial b}p_{j}^{i+1} & \frac{\partial R}{\partial c}p_{j}^{i+1} & I_{3\times 3} \end{array}\right],$$

(63) $$L=\left[\begin{array}{c} l_{1}\\ \vdots \\ l_{k} \end{array}\right],\;l_{j}=p_{j}^{i}-R_{0}p_{j}^{i+1}-T_{0};$$

Step 9: If $j_{0}=j_{0}+1$, go to Step 4;

Step 10: Calculate the root mean square errors of transformation and rotation parameters [3]:

(64) $$RMSE_{r}=\sqrt{\frac{1}{1000}\sum_{j=1}^{1000}\left[{\left({\hat{r}}_{a,j}-{\tilde{r}}_{a}\right)}^{2}+{\left({\hat{r}}_{b,j}-{\tilde{r}}_{b}\right)}^{2}+{\left({\hat{r}}_{c,j}-{\tilde{r}}_{c}\right)}^{2}\right]},$$

(65) $$RMSE_{t}=\sqrt{\frac{1}{1000}\sum_{j=1}^{1000}\left[{\left({\hat{t}}_{x,j}-{\tilde{t}}_{x}\right)}^{2}+{\left({\hat{t}}_{y,j}-{\tilde{t}}_{y}\right)}^{2}+{\left({\hat{t}}_{z,j}-{\tilde{t}}_{z}\right)}^{2}\right]}.$$

### 4.2. Experiment I

**Figure 1:**
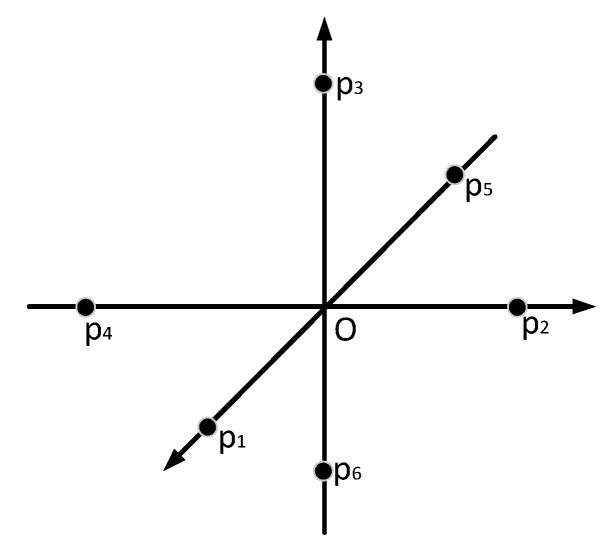
The target geometry of experiment I.

Since the precision of target-based registration is related to the number of targets, we simulated six targets (see Figure 1) and designed four scenarios: **Case A**: using three targets $\left\{{\tilde{p}}_{1}^{i},{\tilde{p}}_{2}^{i},{\tilde{p}}_{3}^{i}\right\}$; **Case B**: using four targets $\left\{{\tilde{p}}_{1}^{i},{\tilde{p}}_{2}^{i},{\tilde{p}}_{3}^{i},{\tilde{p}}_{4}^{i}\right\}$; **Case C**: using five targets $\left\{{\tilde{p}}_{1}^{i},{\tilde{p}}_{2}^{i},{\tilde{p}}_{3}^{i},{\tilde{p}}_{4}^{i},{\tilde{p}}_{5}^{i}\right\}$; and Case D: using six targets $\left\{{\tilde{p}}_{1}^{i},{\tilde{p}}_{2}^{i},{\tilde{p}}_{3}^{i},{\tilde{p}}_{4}^{i},{\tilde{p}}_{5}^{i},{\tilde{p}}_{6}^{i}\right\}$). We then calculated the rDOP, tDOP, $RMSE_{r}$, and $RMSE_{t}$ of Case A, B, C, and D with different target distributions and locations of scanners. The specific experimental processes are as follows:

Step 1: Assume $T_{0}=\left[\begin{array}{c} 0\\ 0\\ 0 \end{array}\right]$ and the coordinate true values of targets are

(66) $${\tilde{p}}_{1}^{i}=\left[\begin{array}{c} 10\\ 0\\ 0 \end{array}\right],\;{\tilde{p}}_{2}^{i}=\left[\begin{array}{c} 0\\ 10\\ 0 \end{array}\right],\;{\tilde{p}}_{3}^{i}=\left[\begin{array}{c} 0\\ 0\\ 10 \end{array}\right],\;{\tilde{p}}_{4}^{i}=\left[\begin{array}{c} 0\\ -10\\ 0 \end{array}\right],\;{\tilde{p}}_{5}^{i}=\left[\begin{array}{c} -10\\ 0\\ 0 \end{array}\right],\;{\tilde{p}}_{6}^{i}=\left[\begin{array}{c} 0\\ 0\\ -10 \end{array}\right];$$

Step 2: Let ${\tilde{p}}_{1}^{i}(1,1)=10+2j_{1}$ and $j_{1}\in \left\{0,1,\cdots ,25\right\}$. Calculate $rDOP_{1}$, $tDOP_{1}$, $RMSE_{r,1}$, and $RMSE_{t,1}$ of Case A, B, C, and D with different locations of target ${\tilde{p}}_{1}^{i}$ from Equations (19), (20), (24), (25), (64) and (65) (see Figure 3);

Step 3: Let ${\tilde{p}}_{1}^{i}(1,1)=10+2j_{1}$, ${\tilde{p}}_{2}^{i}(2,1)=10+2j_{1}$, and $j_{1}\in \left\{0,1,\cdots ,25\right\}$. Calculate $rDOP_{2}$, $tDOP_{2}$, $RMSE_{r,2}$, and $RMSE_{t,2}$ of Case A, B, C, and D with different locations of targets ${\tilde{p}}_{1}^{i}$ and ${\tilde{p}}_{2}^{i}$ from Equations (19), (20), (24), (25), (64) and (65) (see Figure 4);

Step 4: Let ${\tilde{p}}_{1}^{i}(1,1)=10+2j_{1}$, ${\tilde{p}}_{2}^{i}(2,1)=10+2j_{1}$, ${\tilde{p}}_{3}^{i}(3,1)=10+2j_{1}$, and $j_{1}\in \left\{0,1,\cdots ,25\right\}$. Calculate $rDOP_{3}$, $tDOP_{3}$, $RMSE_{r,3}$, and $RMSE_{t,3}$ of Case A, B, C, and D with different locations of targets ${\tilde{p}}_{1}^{i}$, ${\tilde{p}}_{2}^{i}$, and ${\tilde{p}}_{3}^{i}$ from Equations (19), (20), (24), (25), (64) and (65) (see Figure 5);

Step 5: Let $T_{0}=\left[\begin{array}{c} -j_{1}\\ -j_{1}\\ -j_{1} \end{array}\right]$ and $j_{1}\in \left\{0,1,\cdots ,100\right\}$. Calculate $rDOP_{4}$, $tDOP_{4}$, $RMSE_{r,4}$, and $RMSE_{t,4}$ of Case A, B, C, and D with different locations of scanner $T_{0}$ from Equations (19), (20), (24), (25), (64) and (65) (see Figure 6).

### 4.3. Experiment II

**Figure 2:**
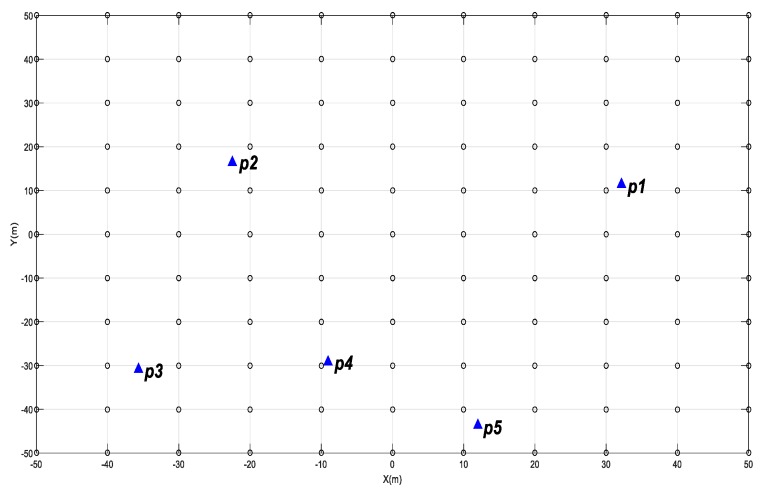
The target and ***scanner i* + 1** geometry of experiment II.

To further verify the quantitative evaluation model of the targets’ geometry distribution, we designed another experiment with realistic targets drawn from previous studies [3,23] using an RIEGL VZ-400 laser scanner with different target distributions (see Table 1) and different locations of scanners (see Figure 2). The specific experimental processes are as follows:

Step 1: Assume the locations of scanner $T_{0}=\left[\begin{array}{c} t_{x}\\ t_{y}\\ -10 \end{array}\right]$, $t_{x},t_{y}\in \left\{-50,-40,\cdots ,40,50\right\}$;

Step 2: Input the coordinates of targets:

(67) $${\tilde{p}}_{1}=\left[\begin{array}{c} 32.135\\ 11.435\\ 0.076 \end{array}\right],\;{\tilde{p}}_{2}=\left[\begin{array}{c} -22.478\\ 16.356\\ 0.127 \end{array}\right],\;{\tilde{p}}_{3}=\left[\begin{array}{c} -35.665\\ -30.837\\ -0.494 \end{array}\right],\;{\tilde{p}}_{4}=\left[\begin{array}{c} -9.061\\ -29.255\\ -0.504 \end{array}\right],\;{\tilde{p}}_{5}=\left[\begin{array}{c} 11.995\\ -43.692\\ -0.4 \end{array}\right];$$

Step 3: Calculate $rDOP_{5}$, $tDOP_{5}$, $RMSE_{r,5}$, and $RMSE_{t,5}$ of Case A1 and B1–5 with different locations of scanner $T_{0}$ from Equations (19), (20), (24), (25), (64) and (65) (see Figure 7);

Step 4: Calculate $rDOP_{6}$, $tDOP_{6}$, $RMSE_{r,6}$, and $RMSE_{t,6}$ of Case C1–10 with different locations of scanner $T_{0}$ from Equations (19), (20), (24), (25), (64) and (65) (see Figure 8).

### 4.4. Results Analysis

**Figure 3:**
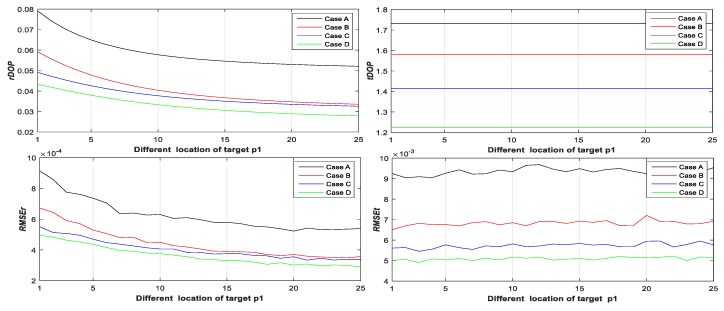
$rDOP_{1}$, $tDOP_{1}$, $RMSE_{r,1}$, and $RMSE_{t,1}$ of Case A, B, C, and D with respect to one different target.

**Figure 4:**
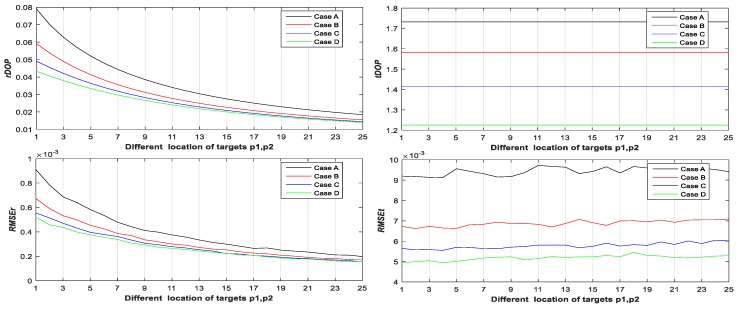
$rDOP_{2}$, $tDOP_{2}$, $RMSE_{r,2}$, and $RMSE_{t,2}$ of Case A, B, C, and D with respect to two different targets.

**Figure 5:**
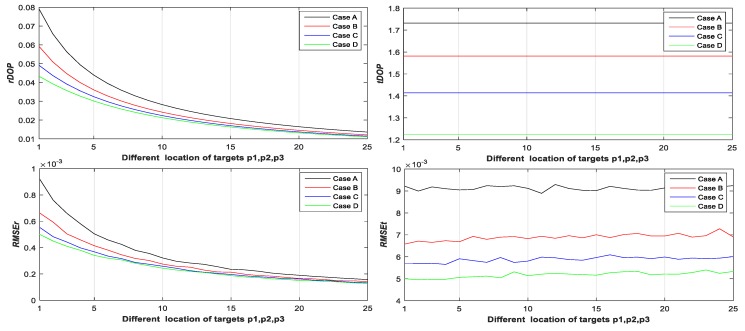
$rDOP_{3}$, $tDOP_{3}$, $RMSE_{r,3}$, and $RMSE_{t,3}$ of Case A, B, C, and D with respect to three different targets.

**Figure 6:**
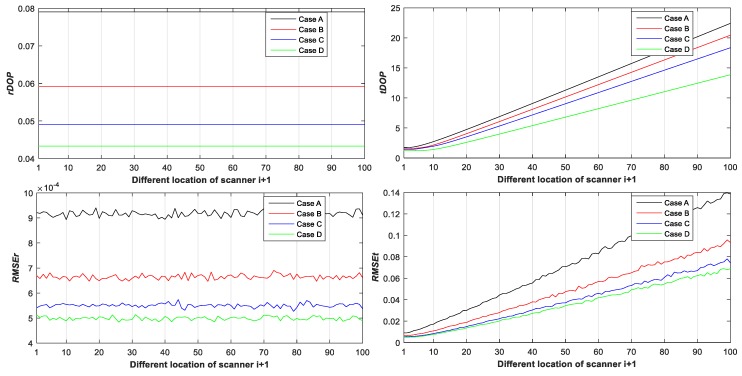
$rDOP_{4}$, $tDOP_{4}$, $RMSE_{r,4}$, and $RMSE_{t,4}$ of Case A, B, C, and D with respect to different ***Scanner i* + 1.**

**Figure 7:**
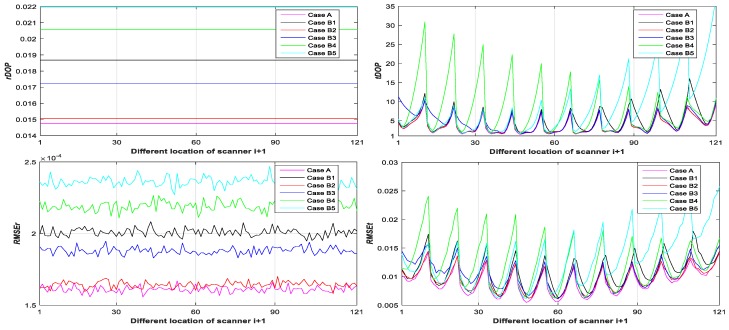
$rDOP_{5}$, $tDOP_{5}$, $RMSE_{r,5}$, and $RMSE_{t,5}$ of Case A and B1–5 with respect to different scanner i + 1.

**Figure 8:**
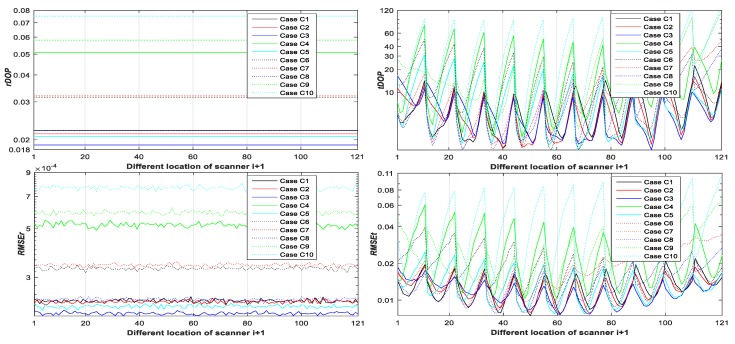
$rDOP_{6}$, $tDOP_{6}$, $RMSE_{r,6}$, and $RMSE_{t,6}$ of Case C1–10 with respect to different ***scanner i* + 1**.

From Figure 3, Figure 4, Figure 5, Figure 6, Figure 7 and Figure 8, it may be concluded that

(a) The change of tDOP is basically the same as the change of $RMSE_{t}$; the size of tDOP and $RMSE_{t}$ is related to the location of ***scanner i* + 1** (see Figure 6, Figure 7 and Figure 8) and the number of targets (see Figure 3, Figure 4 and Figure 5), not the location of targets (see Figure 3, Figure 4 and Figure 5);
(b) The farther away the location of ***scanner i*** **+ 1** (with respect to different $T_{0}$), the greater the tDOP and $RMSE_{t}$ in Figure 6;
(c) When the number and position of targets change, but the location of the scanner is unchanged, the value of tDOP is a constant, the $RMSE_{t}$ is around a constant, and different numbers of targets (with respect to Case A, B, C, and D) have different constant valued of tDOP and $RMSE_{t}$; the more targets (with respect to Case A, B, C, and D), the smaller the tDOP and $RMSE_{t}$ (see Figure 3, Figure 4 and Figure 5);
(d) The change of rDOP is also basically the same as the change of $RMSE_{r}$; the size of rDOP and $RMSE_{r}$ is related to the number and position of targets (see Figure 3, Figure 4 and Figure 5), not the location of ***scanner i* + 1** (see Figure 6, Figure 7 and Figure 8);
(e) The more dispersive the targets (with respect to different locations of targets ${\tilde{p}}_{1}^{i},{\tilde{p}}_{2}^{i},{\tilde{p}}_{3}^{i}$), the smaller the rDOP and $RMSE_{r}$ in Figure 3, Figure 4 and Figure 5;
(f) When the location of the scanner changes, but the number and position of targets are unchanged, the value of rDOP is a constant, the $RMSE_{r}$ is around a constant, and different numbers of targets (with respect to Case A, B, C, D, A1, B1–5, and C1–10) have different constant values of rDOP and $RMSE_{r}$; the more targets there are (with respect to Case A, B, C, D, A1, B1–5, and C1–10), the smaller the rDOP and $RMSE_{r}$ (see Figure 6, Figure 7 and Figure 8);
(g) The differences between the $RMSE_{t}$ and the $RMSE_{t}$ minimum values in cases A1, B1–5, and C1–10 with the minimum tDOP are −0.5, −1.6, −1.8, 0.9, −2, −1.7, −1.5, −1.8, −2.1, −1.5, −1.5, −1.6, −1.3, −1.5, −2.0, and −0.8 mm, respectively, which are all less than $0.5\sigma_{0}$ (half the observation variance, 2.5 mm), so we can use the $RMSE_{t}$ minimum value with the minimum tDOP to represent the $RMSE_{t}$ minimum value;
(h) We can use rDOP and tDOP to assess the impact of the targets’ geometry distribution on the rotation parameters and translation parameters, respectively, and use rDOP and tDOP to help determine the optimal placement location of targets (with respect to the minimum rDOP) and the best location of ***scanner*** ***i*** **+ 1** (with respect to the minimum tDOP).

## 5. Conclusions

This research proposes a new evaluation model of TGD (namely, rDOP and tDOP) for the first time, and quantitatively verifies that the model can be used to assess the impact of TGD on the registration precision by experiments, which show that “the change of rDOP***/***tDOP is basically the same as the change of the registration precision”. In addition, this research also mathematically proves the existing experiences of TGD by the proposed model, such as “The more targets, the higher the registration precision (corresponding to the smaller rDOP and tDOP)”, “The best setting position of the ***Scanner i* + 1** is the barycenter of all targets (corresponding to the minimum tDOP value)”, “The more dispersive the targets, the higher the registration precision (corresponding to the smaller rDOP values)”, “The targets will be not too close to a straight line where bigger rDOP exists”, and “The targets will be not too close on the same plane where bigger tDOP exists”.

If the targets are considered as control points or satellites, we can use the model to help design the optimal control network in engineering surveying and geodetic surveying or the optimal satellite constellation in GNSS. Therefore, we conclude that the proposed rDOP and tDOP model can be considered a unified evaluation model of the TGD, control point distribution, and satellite constellation.

However, it should be noted that “we only theoretically analyze the equal weight model of rDOP and tDOP”, “we also do not use the real TLS field data collection and the actual cases to analyze the application effect of the rDOP and tDOP model”, “the experiments do not consider targets’ positioning precision that are affected by many factors (such as the height of scanner/targets, scanning distance, incident angel, material type of targets, etc. [28])”, and “our experiments do not consider other applications such as the engineering surveying, the geodetic surveying, aerial photogrammetry and so on”. In the future, we will conduct more experiments and simulations to verify our model’s applications.

## Figures and Tables

**Table 1:** Different target distributions.

The Name of Distribution	Including Targets	The Name of Distribution	Including Targets
**Case A1**	$p_{1}$, $p_{2}$, $p_{3}$, $p_{4}$, $p_{5}$	**Case C1**	$p_{1}$, $p_{2}$, $p_{3}$
**Case B1**	$p_{1}$, $p_{2}$, $p_{3}$, $p_{4}$	**Case C2**	$p_{1}$, $p_{2}$, $p_{4}$
**Case B2**	$p_{1}$, $p_{2}$, $p_{3}$, $p_{5}$	**Case C3**	$p_{1}$, $p_{2}$, $p_{5}$
**Case B3**	$p_{1}$, $p_{2}$, $p_{4}$, $p_{5}$	**Case C4**	$p_{1}$, $p_{3}$, $p_{4}$
**Case B4**	$p_{1}$, $p_{3}$, $p_{4}$, $p_{5}$	**Case C5**	$p_{1}$, $p_{3}$, $p_{5}$
**Case B5**	$p_{2}$, $p_{3}$, $p_{4}$, $p_{5}$	**Case C6**	$p_{1}$, $p_{4}$, $p_{5}$
		**Case C7**	$p_{2}$, $p_{3}$, $p_{4}$
		**Case C8**	$p_{2}$, $p_{3}$, $p_{5}$
		**Case C9**	$p_{2}$, $p_{4}$, $p_{5}$
		**Case C10**	$p_{3}$, $p_{4}$, $p_{5}$
